# *Plasmodium falciparum* infection and disease in infancy associated with increased risk of malaria and anaemia in childhood

**DOI:** 10.1186/s12936-023-04646-8

**Published:** 2023-07-26

**Authors:** Liana R. Andronescu, Andrea G. Buchwald, Ankur Sharma, Andy Bauleni, Patricia Mawindo, Yuanyuan Liang, Julie R. Gutman, Don P. Mathanga, Jobiba Chinkhumba, Miriam K. Laufer

**Affiliations:** 1 Center for Vaccine Development and Global Health, University of Maryland School of Medicine, Baltimore, 21201 USA; 2 Malaria Alert Center, Kamuzu University of Health Sciences, Blantyre, Malawi; 3 Department of Epidemiology and Public Health, University of Maryland School of Medicine, Baltimore, 21201 USA; 4 Malaria Branch, Centers for Disease Control and Prevention, Atlanta, 30333 USA

**Keywords:** Malaria, Infants, Incidence, Hemoglobin, Infant development

## Abstract

**Background:**

Infants under 6 months of age are often excluded from malaria surveillance and observational studies. The impact of malaria during early infancy on health later in childhood remains unknown.

**Methods:**

Infants from two birth cohorts in Malawi were monitored at quarterly intervals and whenever they were ill from birth through 24 months for *Plasmodium falciparum* infections and clinical malaria. Poisson regression and linear mixed effects models measured the effect of exposure to malaria in infancy on subsequent malaria incidence, weight-for-age z-scores (WAZ), and haemoglobin concentrations after 6 months.

**Results:**

Infants with at least one *P. falciparum* infection during their first 6 months had increased incidence ratio (IRR) of *P. falciparum* infection (IRR = 1.27, 95% CI, 1.06–1.52) and clinical malaria (IRR = 2.37, 95% CI, 2.02–2.80) compared to infants without infection. Infants with clinical malaria had increased risk of *P. falciparum* infection incidence between 6 and 24 months (IRR = 1.64, 95% CI, 1.38–1.94) and clinical malaria (IRR = 1.85, 95% CI, 1.48–2.32). Exposure to malaria was associated with lower WAZ over time (p = 0.02) and lower haemoglobin levels than unexposed infants at every time interval (p = 0.02).

**Conclusions:**

Infants experiencing malaria infection or clinical malaria are at increased risk of subsequent infection and disease, have poorer growth, and lower haemoglobin concentrations.

## Background

The burden of *Plasmodium falciparum* infection and clinical malaria disease remains highest among under-five children in many regions of sub-Saharan Africa. In these settings, children aged 6 months to 2 years may have up to five episodes of clinical malaria per year [1, 2]. Infants under 6 months of age are typically excluded from observational studies because infants are not considered high risk for malaria due to presumed protective effects from maternal antibodies transferred through the placenta, lactoferrin from breastmilk, and fetal haemoglobin [3, 4]. However, recent facility-based surveys suggest that *P. falciparum* infection and clinical malaria is more common than expected in the under 6-months population in sub-Saharan Africa [5–7]. Associations between malaria and health outcomes, such as anaemia and growth have been studied in older children but the long-term, cumulative impact of early infections have not been well-characterized.

Malaria exposure in infancy may impact the health of young children, beyond the morbidity of acute infection. Infections of all varieties are known to impair nutritional status resulting in growth faltering, however the role of early *P. falciparum* infections in growth and development remains poorly defined and the literature is inconclusive [8–10]. Malaria infection is a leading cause of anaemia among young children in endemic regions of sub-Saharan Africa [11, 12]. *Plasmodium falciparum* infection causes both red blood cell destruction and reduced production), resulting in low haemoglobin levels. The association is strong enough that anaemia prevalence has been proposed as an indicator of acute malaria prevalence in malaria endemic regions, though less is understood about the impact on subsequent anaemia in early childhood [13–15]. Malaria infection is associated with low haemoglobin right after infection, but cumulative effects of repeated *P. falciparum* infections are not well documented in infants, although there is evidence of an association between *Plasmodium vivax* and anaemia in infants [16].

The study was designed to use longitudinal data collected from two birth cohorts in Malawi to measure malaria infection and disease in early infancy and the association this exposure has with subsequent malaria incidence in early childhood. Because malaria infection during early infancy may enhance or interfere with immunity and subsequent clinical response to malaria infection, the study tested the hypothesis that clinical malaria in the first 6 months of life would be associated with a statistically significant increase in incidence of *P. falciparum* infection and clinical malaria during early childhood. The study further evaluated the hypothesis that infants with *P. falciparum* infection or clinical malaria in the first 6 months may have lower weight-for-age z-scores and lower haemoglobin concentrations over time compared to unexposed infants.

## Methods

### Participants

Study participants from two prospective cohorts conducted in southern Malawi were included in this study because they were conducted during the same timeframe and with similar follow-up procedures. The Liwonde cohort enrolled infants born to mothers who participated in a clinical trial that randomly allocated 602 pregnant women to receive monthly administration of either sulfadoxine-pyrimethamine or dihydroartemisinin-piperaquine as intermittent preventive treatment during pregnancy (NCT03009526). Women aged 16 years or older, with a viable singleton pregnancy at less than or equal to 28 gestational weeks were included in the clinical trial if they were HIV-negative and were not experiencing a high-risk pregnancy or experiencing severe anaemia. During pregnancy, women were offered the opportunity to enroll their babies in the infant cohort after completing the clinical trial. Inclusion criteria for mothers enrolling their babies in the infant cohort included intention to stay in the study area for at least one year following delivery and provision of informed consent. Participant enrollment in the infant cohort began in May 2017 and follow up was completed in May 2019. Details of this cohort were described previously [17].

The Mfera cohort enrolled 100 infants at delivery. Infant’s mothers were recruited for the study at the antenatal clinic or the well-child clinic. Enrollment criteria included: provision of informed consent by the parent or guardian, infant born to a mother with documented HIV-negative status, intention to reside in the study region for two years, and willingness to comply with study procedures and attend the health center for regular medical care. Enrollment for the Mfera cohort ended in January 2016 and the final participant completed follow-up in November 2018.

Both studies were reviewed and approved by the University of Maryland Institutional Review Board and the Kamuzu University of Health Sciences College of Medicine Research Ethics Committee. Parents/guardians provided written informed consent for participation of the infant.

### Study procedures

All women in the Liwonde clinical trial delivered in the maternity wards at Machinga District Hospital in Liwonde, while infants in the Mfera cohort were born at the Mfera Health Centre in Chikwawa, both in southern Malawi. Data collected at enrollment included demographic characteristics and pregnancy history, including infant sex, maternal age, gravidity, and parity.

Follow-up procedures were the same in both cohorts. Infants visited their respective study clinics for scheduled visits at 3-month intervals for up to 24 months. Growth measurements including weight, length, and head circumference were taken at each scheduled visit. Haemoglobin concentration was only measured in infants enrolled in the Mfera cohort. Mothers were encouraged to bring infants to the clinic in the event of illness. If an infant presented with signs and symptoms consistent with malaria at any visit (axillary fever of > 37.5 °C, report of fever in the previous 48 h, vomiting, irritability or weakness), a Paracheck-Pf (Orchid Biomedical Systems, Goa, India) rapid diagnostic test (RDT) for malaria was performed. All cases of malaria detected by RDT were treated with artemether-lumefantrine according to the national treatment policy in Malawi. Dried blood spots on 3M Whatman filter paper were collected at every scheduled and unscheduled visit from a finger prick.

### Laboratory procedures

Asymptomatic and submicroscopic infections were detected from the dried blood spots collected at every visit. Dried blood spots underwent DNA extraction and real time quantitative polymerase chain reaction (qPCR) at the University of Maryland malaria laboratory for detection of the 18S ribosomal RNA gene of *P. falciparum* [18, 19]. The qPCR results were delayed and thus not available to participants.

### Definitions

Exposure to malaria in the first 6 months of life was assessed and defined three ways:

A) Asymptomatic *P. falciparum* infection includes infants with a positive qPCR but excludes infants with signs and symptoms of malaria accompanied by a positive RDT
B) Clinical malaria was defined as signs or symptoms of malaria recorded at the clinic visit and a positive RDT, and
C) All *P. falciparum* infections include all infants with clinical malaria or a positive qPCR.

Three outcomes were measured. Incidence of any malaria, including both asymptomatic *P. falciparum* infection and clinical malaria cases, and incidence of clinical malaria between 6 months of age and the end of follow-up (up to 2 years of age) were measured by the total number of malaria cases divided by the number of person-years at risk. Mean weight-for-age Z-score (WAZ) was assessed at three-month intervals during follow-up (6–24 months) classified according to WHO child growth standards. Mean haemoglobin concentration was assessed at three-month intervals during follow-up (6–24 months) in the Mfera cohort only.

Season of birth was classified as rainy (December–March) or dry (April–November) using historical definitions of rainy and dry seasons in the region. Length of follow-up times varied for participants, so an additional measure of seasonal exposure was included that measured the percent of study time each infant spent in the rainy season. A covariate measured malaria exposure during the follow-up period in the longitudinal analyses as a cumulative count of all malaria infections per individual.

### Statistical analysis

Infants with follow up that ended prior to 9 months of age were excluded from these analyses due to inadequate follow up time. Covariates assessed include infant sex, study site, season of birth, percent of follow-up time in the rainy season, low birth weight (< 2.5 kg), maternal age at delivery, and maternal gravidity. Although data from the two studies were combined, study site was included because of varying environmental factors between the two sites. Maternal age at delivery and gravidity were assessed continuously. Bivariate analysis using Chi-square tests and Student’s t-tests were conducted to describe participant characteristics.

Poisson regression was employed to estimate the incidence rate of exposure to malaria in the first 6 months of life and the incidence rate ratio (IRR) for malaria up to 24 months of age comparing those with and without malaria exposure in the first 6 months of life. To adjust for the effect of covariates on the association between the predictors and outcomes, multivariable Poisson regression models were developed with offset of follow up time to control for varying infection and disease rates.

Unadjusted mean WAZ and haemoglobin were plotted over time and adjusted linear mixed effects models, with participant as the random effect, were built and graphed. An interaction term of exposure and infant age was included in every model, regardless of statistical significance, to visualize the difference in slope between the exposed and unexposed infants.

All analyses were conducted using SAS software, Version 9.4 of the SAS System for Microsoft Windows.

## Results

### Baseline characteristics

A total of 437 infants with complete data and sufficient follow-up time were included in the final analyses (Table 1). Infant sex, maternal gravidity, and maternal age were not differentially distributed in any of the exposure categories. The proportion of infants with clinical malaria in the first 6 months of life was significantly higher in the Mfera cohort (p =  < 0.0001) compared to participants in the Liwonde cohort, but the proportion of infants with asymptomatic *P. falciparum* infection was not different. While infants in the Mfera cohort comprised 21% of the total study population, they made up 47% of the infants with clinical malaria.

**Table 1:** Characteristics of infants at birth and cumulative *P. falciparum* infections after 6 months of age

Characteristics of infants and mothers in Liwonde and Mfera cohorts											
		All (N = 437)	Any malaria infection			Asymptomatic *P. falciparum* infection			Clinical malaria		
			Yes (n = 127)	No (n = 310)	P-value	Yes (n = 99)	No (n = 289)	P-value	Yes (n = 49)	No (n = 388)	P-value
Infant sex^a^	Male	195 (45)	58 (46)	137 (45)	0.79^b^	43 (44)	124 (43)	0.93^b^	28 (57)	167 (43)	0.07^b^
Study site	Liwonde Mfera	344 (79) 93 (21)	93 (73) 34 (27)	251 (81) 59 (19)	0.07^b^	81 (82) 18 (18)	237 (82) 52 (18)	0.97^b^	26 (53) 23 (47)	318 (82) 70 (18)	< .0001^b^
Season of birth	Rainy	146 (33)	55 (43)	91 (29)	0.005^b^	43 (43)	85 (29)	0.01^b^	18 (37)	128 (33)	0.60^b^
Maternal gravidity	Mean (SD)	1.9 (1.8)	2.1 (1.9)	1.8 (1.7)	0.08^c^	1.9 (1.8)	1.8 (1.7)	0.44^c^	2.2 (2.1)	1.8 (1.7)	0.21^c^
Maternal age (years)	Mean (SD)	25 (6)	26 (7)	25 (6)	0.05^c^	26 (7)	25 (6)	0.06^c^	25 (7)	25 (6)	0.82^c^
Rainy season (%)	Mean (SD)	53 (7)	52 (7)	54 (7)	0.0009^c^	52 (7)	54 (7)	0.005^c^	52 (7)	54 (7)	0.19^c^
Low birthweight (< 2.5 kg)	Yes	34 (8)	9 (7)	25 (8)	0.73^b^	7 (7)	23 (8)	0.78^b^	4 (8)	30 (8)	0.92^b^

Characteristics of infants and mothers in the Mfera cohort only											
		All (N = 93)	Yes (n = 39)	No (n = 54)	P-value^a^	Yes (n = 20)	No (n = 50)	P-value^a^	Yes (n = 23)	No (n = 70)	P-value^a^
Infant sex	Male	42 (45)	19 (49)	23 (43)	0.56	9 (45)	21 (42)	0.82	12 (52)	30 (43)	0.44
Season of birth	Rainy	25 (27)	16 (41)	9 (17)	0.009	8 (40)	9 (18)	0.05	8 (35)	17 (24)	0.32
Maternal gravidity	Mean (SD)	2.6 (2.1)	2.7 (2.1)	2.5 (2.1)	0.56^c^	2.7 (1.8)	2.6 (2.1)	0.89^c^	2.6 (2.4)	2.6 (2.5)	0.95^c^
Maternal age in years, mean (SD**)**		26 (7)	27 (6)	26 (7)	0.61^b^	27 (6)	26 (7)	0.79^b^	26 (7)	26 (7)	0.76^b^

^a^Missing 4 infant sex values; ^b^Chi-square; ^c^Student’s t-test

### Malaria incidence in infants

Among 127 (27%) infants in the first 6 months of life, there were 241 episodes of any malaria, with an incidence rate of 1.1 episodes per person-year and 69 episodes of clinical malaria with an incidence rate of 0.3 cases per person-year, all in the first 6 months of life. Among 99 (26%) infants with asymptomatic *P. falciparum* infections in the first 6 months of life, the incidence of any *P. falciparum* infections after 6 months of life was 2 cases per person-year and incidence of clinical malaria after 6 months of life was 0.9 cases per person-year. Among the 49 (11%) infants with clinical malaria in the first 6 months of life, incidence of asymptomatic *P. falciparum* infections was 3.8 cases per person-year and incidence of clinical malaria was 2.3 cases per person-year after the first 6 months of life. Results are summarized in Table 2.

**Table 2:** Incidence rate ratio of any malaria and clinical malaria between 6 and 24 months and mean cumulative episodes of malaria at follow-up timepoints by cohort

	N = 437	Follow-up (person years)	Any malaria infections				Clinical malaria			
			Cases	Incidence PPY^a^	Unadjusted IRR (95% CI)	P-value^b^	Cases	Incidence PPY^a^	Unadjusted IRR (95% CI)	P-value^b^
Any malaria (first 6 months)										
No	310	279.1	439	1.57	REF	< .0001	210	0.75	REF	< 0.0001
Yes	127	114.6	303	2.64	1.68 (1.45–1.95)		161	1.40	1.87 (1.52–2.29)	
Asympomatic. *Pf* infection (first 6 months)^c^										
No	289	259.8	381	1.47	REF	0.0009	179	0.69	REF	0.06
Yes	99	81.9	164	2.00	1.37 (1.14–1.64)		73	0.89	1.29 (0.98–1.70)	
Clinical malaria (first 6 months)										
No	388	341.7	545	1.59	REF	< .0001	252	0.74	REF	< 0.0001
Yes	49	52.0	197	3.79	2.37 (2.02–2.80)		119	2.29	3.10 (2.49–3.87)	
Infant sex										
Female	238	209.4	389	1.86	REF	0.76	186	0.89	REF	0.21
Male	195	182.0	346	1.90	1.02 (0.88–1.18)		184	1.01	1.14 (0.93–1.40)	
Study site										
Liwonde	344	260.7	258	0.99	REF	< .0001	76	0.29	REF	< 0.0001
Mfera	93	133.0	484	3.64	3.68 (3.16–4.28)		295	2.22	7.61 (5.91–9.79)	
Season of birth										
Dry	146	262.4	529	2.02	REF	0.007	279	1.06	REF	0.0005
Rainy	291	131.3	213	1.62	0.80 (0.69–0.94)		92	0.70	0.66 (0.52–0.83)	
% follow-up time in rainy season										
Each unit increase	437	393.7	742	1.88	0.95 (0.94- 0.96)	< .0001	371		0.94 (0.93–0.94)	< 0.0001
Maternal age										
Each year increase	437	393.7	742	1.88	0.98 (0.97–0.99)	0.001	371	0.94	0.97(0.95–0.98)	0.0002
Gravidity										
Each unit increase	437	393.7	742	1.88	1.05 (1.01–1.09)	0.01	371	0.94	1.04 (0.98–1.09)	0.21

^a^Per person-year; ^b^Chi-square; ^c^Sample restricted to participants who did not experience clinical malaria during first 6 months

In multivariable analysis controlling for study site, season of birth, maternal age, and gravidity, incidence of any malaria up to 24 months of age among infants with any *P. falciparum* infection during their first 6 months was 27% higher than unexposed infants (IRR = 1.27, 95% CI, 1.06–1.52, Table 3) and clinical malaria incidence was 76% higher in exposed infants (IRR = 1.76, 95% CI, 1.42–2.19). Any malaria incidence among infants who experience clinical malaria during the first 6 months was 64% higher than unexposed infants (IRR = 1.64, 95% CI, 1.38–1.94) and clinical malaria incidence was 85% higher in exposed infants (IRR = 1.85, 95% CI, 1.48–2.32).

**Table 3:** Multivariable associations between malaria exposure in the first 6 months and incidence of malaria after 6 months of age

	Any malaria incidence		Clinical malaria incidence	
	Adjusted IRR (95% CI)	P-value	Adjusted IRR (95% CI)	P-value
*Any malaria exposure*				
Any malaria				
None	REF	0.009	REF	< 0.0001
Malaria	1.27 (1.06–1.52)		1.76 (1.42–2.19)	
Study site				
Liwonde	REF	< 0.0001	REF	< 0.0001
Mfera	3.52 (2.83–3.90)		6.98 (5.37–9.08)	
Season of birth				
Dry	REF	0.03	REF	0.0002
Rainy	0.84 (0.71–0.99)		0.72 (0.57–0.92)	
Maternal age				
Per unit increase	0.94 (0.92–0.96)	< 0.0001	0.92 (0.90–0.95)	< 0.0001
Gravidity				
Per unit increase	1.13 (1.05–1.20)	< .0.0001	1.13 (1.04–1.22)	0.005
*Asymptomatic P. falciparum exposure*				
Asymp. *Pf* infection				
None	REF	< 0.0001	REF	0.002
*Pf* infection	1.53 (1.26–1.85)		1.56 (1.18–2.07)	
Study site				
Liwonde	REF	< 0.0001	REF	< 0.0001
Mfera	3.24 (2.71–3.89)		7.41 (5.44–10.07)	
Season of birth				
Dry	REF	0.001	REF	0.0004
Rainy	0.71 (0.58–0.87)		0.55 (0.40–0.77)	
Maternal age				
Per unit increase	0.95 (0.93–0.97)	< 0.0001	0.92 (0.89–0.95)	< 0.0001
Gravidity				
Per unit increase	1.13 (1.05–1.22)	0.001	1.19 (1.07–1.31)	0.0009
*Clinical malaria exposure*				
Clinical malaria				
None	REF	< 0.0001	REF	< 0.0001
Clinical malaria	1.64 (1.38–1.94)		1.85 (1.48–2.32)	
Study site				
Liwonde	REF	< 0.0001	REF	< 0.0001
Mfera	3.19 (2.71–3.75)		6.58 (5.04–8.60)	
Season of birth				
Dry	REF	0.02	REF	0.002
Rainy	0.83 (0.70–0.97)		0.69 (0.54–0.87)	
Maternal age				
Per unit increase	0.95 (0.93–0.96)	< 0.0001	0.93 (0.90–0.95)	< 0.0001
Gravidity				
Per unit increase	1.11 (1.05–1.18)	0.0005	1.12 (1.03–1.22)	0.006

^a^Poisson regression

### Effect of malaria exposure on growth

**Fig. 1:**
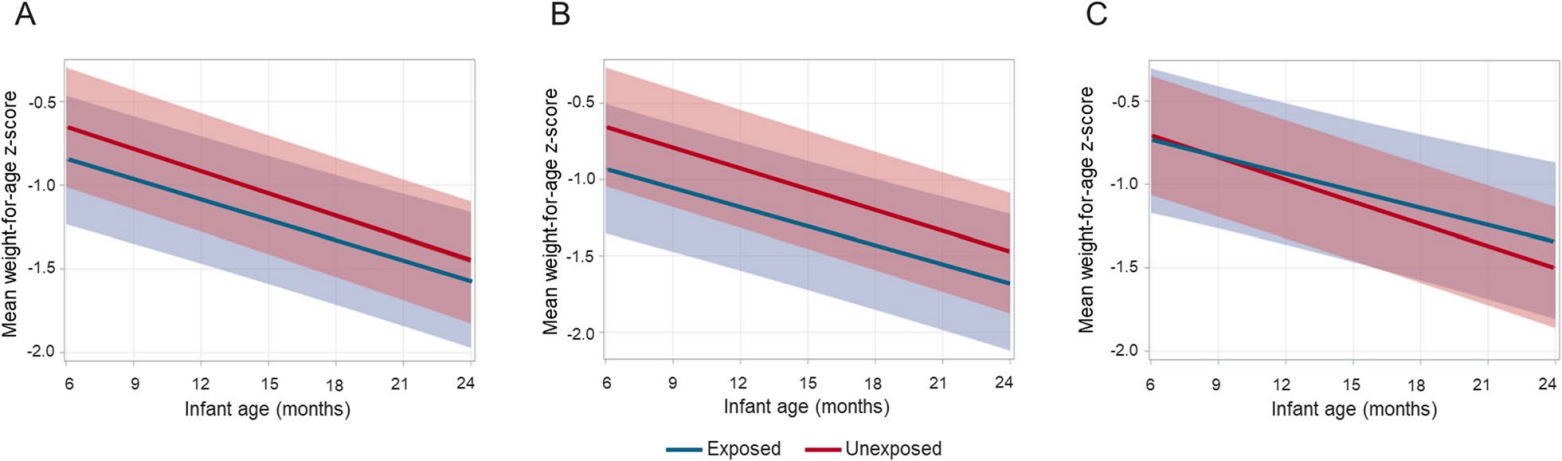
**A** WAZ over follow-up time by any malaria exposure adjusted for infant age, infant sex, low birthweight, and an interaction term of infants age and malaria exposure (p = 0.21); **B** WAZ over follow-up time by asymptomatic *P. falciparum* infection exposure adjusted for infant age, infant sex, low birthweight, and an interaction term of infants age and malaria exposure (p = 0.34); **C** WAZ over follow-up time by clinical malaria exposure adjusted for infant age, infant sex, low birthweight, and an interaction term of infants age and malaria exposure (p = 0.08). The shaded area indicated 95% confidence intervals

The unadjusted mean WAZ for exposed and unexposed infants in all three analyses were between -0.2 and -1.2 (Fig. 1). After including an interaction term comprising infant age and exposure status (to assess the slope of WAZ over the course of follow-up) and adjusting for infant age, malaria during follow-up, infant sex, and low birthweight, the difference in WAZ between infants exposed to any malaria before 6 months and unexposed infants was -0.21 for the duration of follow-up (p = 0.06, Table 4). The difference in WAZ between infants exposed to asymptomatic *P. falciparum* infection before 6 months and unexposed infants was -0.29 for the duration of follow-up (p = 0.02). Across all 3 analyses, mean WAZ decreased over time in both the exposed and unexposed infants, but never fell to more than two standard deviations below the mean (Fig. 1)

.

**Table 4:** Adjusted^a,b^ linear continuous mixed effects models by malaria exposure before 6 months

	Weight-for-age Z-score (WAZ) (n = 429)		Haemoglobin concentration (n = 93)	
	Difference in WAZ	P-value	Difference in haemoglobin (g/dL)	P-value
*Any malaria infection*				
Exposed	-0.21	0.06	-0.38	0.09
Infant age*exposed	0.02	0.41	-0.01	0.89
Infant age	-0.13	< 0.0001	0.30	< 0.0001
Malaria during follow-up	0.02	0.08	-0.05	0.01
Male	-0.24	0.01	–	–
Low birth weight (< 2.5 kg)	-0.50	0.01	–	–
*Asymptomatic P. falciparum infection*				
Exposed	-0.29	0.02	-0.26	0.34
Infant age*exposed	0.01	0.47	-0.07	0.18
Infant age	-0.14	< 0.0001	0.28	< 0.0001
Malaria during follow-up	0.02	0.14	-0.03	0.18
Male	-0.27	0.01	–	–
Low birth weight (< 2.5 kg)	-0.53	0.01	–	–
*Clinical malaria*				
Exposed	-0.06	0.69	-0.57	0.03
Infant age*exposed	0.03	0.08	0.09	009
Infant age	-0.13	< 0.0001	0.29	< 0.0001
Malaria during follow-up	0.01	0.20	-0.07	0.01
Male	0.25	0.01	–	–
Low birth weight (< 2.5 kg)	-0.49	0.01	–	–

^a^Models measuring WAZ included an interaction term for infant age and exposure adjusted for malaria during follow-up, sex, and low birth weight

^b^ Models measuring haemoglobin included an interaction term for infant age and exposure and adjusted for malaria during follow-up

### Effect of malaria exposure on haemoglobin concentration

**Fig. 2:**
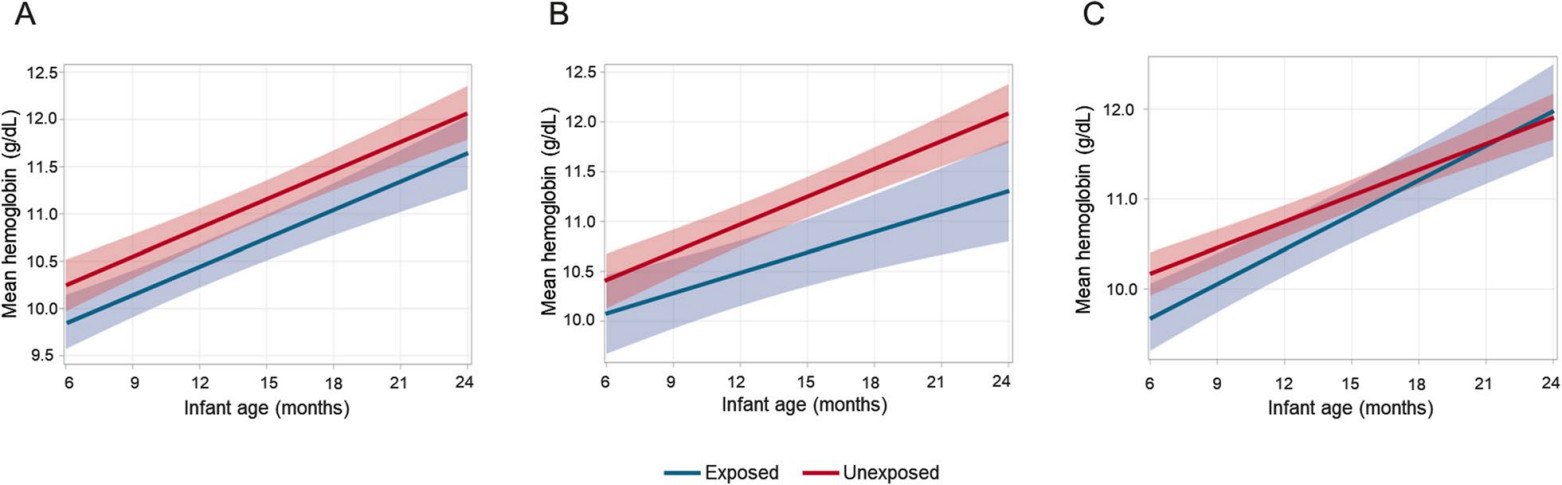
**A** Hemoglobin concentration over follow-up time by any malaria exposure adjusted for infant age, any malaria during follow-up, and an interaction term of infants age and malaria exposure (p = 0.41); **B** Hemoglobin concentration over follow-up time by asymptomatic *P. falciparum* infection exposure adjusted for infant age, any malaria during follow-up, and an interaction term of infants age and malaria exposure (p = 0.12); **C** Hemoglobin concentration over follow-up time by clinical malaria exposure adjusted for infant age, any malaria during follow-up, and an interaction term of infants age and malaria exposure (p = 0.33). The shaded area indicated 95% confidence intervals

Unadjusted mean haemoglobin concentrations for exposed and unexposed infants were below 11 g/dL from 6 months of age until at least 12 months of age, indicating mild to moderate anaemia in the first year of life (Fig. 2). After including an interaction term comprising infant age and exposure status to look at the differences in slope, and adjusting for infant age and malaria during follow-up, the difference in haemoglobin concentration between infants exposed to any malaria before 6 months and unexposed infants was -0.38 g/dL for the duration of follow-up (p = 0.09, Table 4). Mean haemoglobin concentration in infants exposed to asymptomatic *P. falciparum* infections differed by -0.26 g/dL compared to unexposed infants at 6 months (p = 0.34) and the difference steadily increased over time so that while the mean haemoglobin levels of unexposed infants reached the normal range (> 11 g/dL) around 12 months, exposed infants only reached that threshold around 21 months. The difference in mean haemoglobin levels between infants exposed to clinical malaria during their first 6 months and unexposed infants was -0.57 g/dL (p = 0.03).

## Discussion

This study found that infection with *P. falciparum* 6 months of life, whether symptomatic or asymptomatic, was associated with higher incidence of both malaria infection and clinical malaria in early childhood. Additionally, any malaria infection during the first 6 months of life is associated with lower haemoglobin levels over time and lower weight-for-age z-scores compared to children uninfected during the first 6 months.

Infants under 6 months of age have historically been excluded from malaria studies and thus the results about the burden of infection and disease in this population is an important contribution to the literature. One-third of infants in our study had at least one episode of clinical malaria or *P. falciparum* infection before reaching 6 months, confirming that infants are susceptible to malaria during early infancy. Nearly twice as many infants had asymptomatic *P. falciparum* infections before 6 months as infants who experienced clinical malaria during that period, which is consistent with other studies demonstrating relatively high rates of parasitaemia and lower rates of symptomatic malaria illness before 6 months [20].

Several possible causes can explain our finding of higher rates of malaria infection and diseases among infants infected with malaria in the first 6 months of life. Acquired immunity to malaria develops after repeated infection with *P. falciparum*, however despite their early infection or even multiple infections, the exposed infants in this study did not experience reduced incidence during follow-up suggesting that acquired immunity does not develop this early in life, mirroring the poor immunogenicity of live viral and certain antibacterial vaccines which do not induce immunity in young children. Alternatively, these higher rates may be a marker of environmental exposure to malaria, as demonstrated by the difference in malaria incidence observed in the study sites, with higher infection and clinical malaria rates in observed in the Mfera cohort, or a result of differential exposure to *P. falciparum *in utero.

Mean WAZ scores in infants infected with clinical malaria in their first 6 months are higher than unexposed infants at 24 months, while these measures are lower in infants with asymptomatic *P. falciparum* infection at 24 months compared to without malaria in their first 6 months. This may have been observed because infants with asymptomatic *P. falciparum* infection were classified as unexposed in the clinical malaria model, thereby raising the risk of infants classified as unexposed when assessing the risk posed by clinical malaria in the first 6 months of life and confirmed this with a sensitivity analysis that excluded infants with asymptomatic *P. falciparum* infection in the first 6 months of life.

Infants with clinical malaria in their first 6 months were diagnosed and treated so that their infections were cleared, whereas infants with asymptomatic infections could have prolonged infections, resulting in chronic infection causing slower growth and lower haemoglobin. This is consistent with recent research showing that asymptomatic malaria infections are prevalent in anaemic children [21].

The analysis identified a trend of decreasing mean weight-for-age z-scores over time in all infants regardless of malaria exposure in their first 6 months. A systematic review of this topic surmised that most studies found no association between malaria exposure and malnutrition (WAZ ≤ − 2.0) among populations in *P. falciparum* endemic areas [22]. However, to our knowledge, there are no comparable studies that assesses the long-term risk of low WAZ following exposure to malaria in the first 6 months and the association between anthropometry and malaria warrants further exploration.

Malaria is a known cause of low haemoglobin; however, this study suggests that infants exposed to *P. falciparum* infections during their first 6 months of life already have low haemoglobin levels at 6 months of age and continue to have lower haemoglobin levels compared to their unexposed peers through 2 years of age. The data analysis approach tried to control for the possibility that this trend may be due to repeated infections during follow-up by adjusting the models for cumulative malaria infections between 6 and 24 months. Malaria infections cause haemoglobin concentrations to drop rapidly after initial infection and it can take 4–6 weeks to completely normalize [11]. Infant haemoglobin levels drop off naturally after birth and only begin to recover around 6 months of age, it is possible that malaria infection and the accompanying slow recovery rate of haemoglobin concentrations during this period may be responsible for the prolonged haemoglobin recovery times observed in this population.

The primary limitation of this study is the inability to control for the confounding of environmental malaria exposure that is associated with both the exposure of interest and infection outcomes. If infants live in an environment with increased likelihood of acquiring malaria infection, they will have high rates of malaria throughout their childhood. This limits the ability to assess a causal relationship between early malaria infection and subsequent burden of infection and may confound our finding of decreased WAZ and haemoglobin after early malaria infection. An additional limitation in this analysis was missing data on socioeconomic status, nutrition, or breastfeeding. Socioeconomic status, in particular, may be a predictor of both malaria risk and food insecurity though the sample population belonged to the same socioeconomic group. Mothers in the Mfera cohort all reported breastfeeding until the end of follow-up, so dietary variation were unlikely to be different between exposed and unexposed infants in the haemoglobin analysis.

## Conclusion

This study demonstrated that infants with malaria infection in the first 6 months of their lives have chronically lower haemoglobin levels than uninfected infants in the same cohort and higher incidence of malaria overall during early childhood. These early malaria episodes do not appear to elicit protective immunity. Rather, malaria infection before 6 months of age may be an indicator of increased environmental exposure risk suggesting a need to implement additional preventative measures targeting the household or neighbourhood, for example, focal indoor residual spraying or provision of chemoprophylaxis to these children. These results demonstrate the long-term impact of early malaria infection exposure on early childhood health and suggest a need for additional public health guidance for monitoring haemoglobin levels in young children with a history of malaria infection.

## Data Availability

The data will be available upon request made to the corresponding author.
